# Study protocol: The DUALITY trial—a register-based, randomized controlled trial to investigate dual mobility cups in hip fracture patients

**DOI:** 10.1080/17453674.2020.1780059

**Published:** 2020-06-22

**Authors:** Olof Wolf, Sebastian Mukka, Maja Notini, Michael Möller, Nils P Hailer, the DUALITY GROUP

**Affiliations:** a Department of Surgical Sciences, Orthopaedics, Uppsala University;; b Department of Surgical and Perioperative Science (Orthopaedics), Umeå University;; c Institute of Clinical Science, Sahlgrenska Academy, University of Gothenburg, Gothenburg, Sweden

## Abstract

Background and purpose — Physically and mentally fit patients with a displaced femoral neck fracture (FNF) are mostly treated with total hip arthroplasty (THA). Dislocation is a severe and frequent complication in this group, and dual mobility cups (DMC) were developed to reduce the risk of dislocation after THA. The DUALITY trial investigates whether the use of DMC in FNF patients treated with a THA reduces the risk of dislocation.

Patients and methods — The trial is a national, multicenter, register-based, randomized controlled trial (rRCT). Patients ≥ 65 years with a non-pathological, displaced FNF (Type Garden 3–4/AO 31-B2 or B3) who are suitable for a THA according to local guidelines are assessed for eligibility using the web-based registration platform of the Swedish Fracture Register (SFR). 1,600 patients will be randomized 1:1 to either insertion of a DMC (intervention group) or a standard cup (control group). The study is pragmatic in that the choice of implant brands, surgical approach, and peri- and postoperative protocols follow the local routines of each participating unit. All outcome variables will be retrieved after linkage of the study cohort obtained from the SFR with the Swedish Hip Arthroplasty Register and the National Patient Register.

Outcomes — The primary outcome is the occurrence of any dislocation of the index joint treated with closed or open reduction within 1 year after surgery, expressed as a relative risk when comparing groups, and a risk reduction of at least 45% is considered clinically relevant. Secondary outcomes are the relative risk of any reoperation of the index joint, periprosthetic joint infection, and mortality within 90 days and 1 year. Patient-reported outcomes and health economics are evaluated.

Start of trial and estimated duration — The DUALITY trial started recruiting patients in January 2020 and will continue for approximately 5 years.

Trial registration — The trial is registered at clinicaltrials.gov (NCT03909815; December 12, 2019).

Most patients with a displaced femoral neck fracture (FNF) are treated with an arthroplasty, and those who are independently mobile, have few comorbidities, and are cognitively intact commonly receive a total hip arthroplasty (THA) rather than a hemiarthroplasty (Bhandari and Swiontkowski 2017). However, FNF patients treated with a THA often suffer from dislocation, which results in long-lasting impairment of quality of life (Enocson et al. 2009a). The incidence of dislocations after THA performed due to FNF is up to 13% (Jobory 2020), thus being much higher than after THA performed due to osteoarthritis (Johansson et al. 2000, Phillips et al. 2003, Meek et al. 2006, Skoldenberg et al. 2010). Most dislocations occur during the first postoperative year (Phillips et al. 2003, Meek et al. 2006, Hailer et al. 2012), and small femoral head sizes, the posterior surgical approach, comorbidity burden, and male sex are all associated with an increased risk of dislocation (Jolles et al. 2002, Phillips et al. 2003, Meek et al. 2006, Conroy et al. 2008, Enocson et al. 2009b, Kim et al. 2009, Hailer et al. 2012, Ko and Hozack 2016).

Dual mobility cups (DMC) were introduced in order to improve joint stability. In the DMC, a spherical polyethylene liner encloses the metal femoral prosthesis head of standard diameter (mostly 22 or 28 mm), and this liner is mobile within an external metal shell in order to increase range of motion and jumping distance (Caton and Ferreira 2017, Cuthbert et al. 2019). According to a systematic review of observational studies on primary and revision THA performed on a multitude of indications the use of DMC is associated with comparatively low dislocation rates (Darrith et al. 2018). A prospective study on FNF patients operated on with DMC reports a relatively low dislocation rate of 1.4% within 9 months (Adam et al. 2012), but without a comparison group. A large-scale observational study on 9,040 FNF patients treated with THA estimates a considerably reduced risk of revision due to dislocation in patients operated on with DMC compared with those receiving standard cups (Jobory et al. 2019), but the low rate of revisions due to dislocation of 0.8% in that study is contradicted by a considerably higher dislocation rate of 4.7% in a smaller observational study (Tabori-Jensen et al. 2019). It must be noted that arthroplasty register-based studies only report revisions due to dislocation, but not the incidence of dislocations per se, such that the true dislocation rate is consistently underestimated in arthroplasty register studies (Devane et al. 2012).

Further to doubts related to the efficacy of the DMC concept there are concerns regarding its safety. The concept of a large polyethylene liner ensheathed between two metal surfaces might increase polyethylene wear (Tabori-Jensen et al. 2018) and the subsequent risk of aseptic loosening (Caton et al. 2014), and an increased risk of periprosthetic joint infections (PJI) is also reported after the use of DMC (Kreipke et al. 2019), although this notion has been contested (Jobory et al. 2019). A design-specific complication of DMC is intra-prosthetic dislocation (Philippot et al. 2013, Darrith et al. 2018). Thus, although observational evidence indicates that DMC confers a reduced risk of dislocation, both the efficacy and safety of DMC in patients with FNF are uncertain, and no high-level evidence study has yet been conducted (Griffin et al. 2016).

The primary aim of this trial is to investigate whether the use of DMC reduces the risk of dislocation after THA surgery performed due to FNF when compared with standard cups. As secondary endpoints we shall analyze whether the risk of the adverse events any reoperation, periprosthetic joint infection, and mortality is increased after use of the DMC, patient-reported outcomes measured are compared, and the question as to whether use of the costlier DMC is cost-efficient will be addressed.

## Patients and methods

### Study design

The DUALITY trial is a multicenter, register-nested, randomized controlled trial (rRCT; James et al. 2015). Patients with a displaced FNF who are eligible for a THA according to local guidelines are randomized 1:1 to intervention (DMC) or control treatment (standard cup).

### Study subjects and eligibility criteria

The SFR study platform detects eligible patients based on age (≥ 65 years) and type of fracture (type 3 or 4 according to Garden [Kazley et al. 2018], AO types 31-B2 or B3) during registration of the injured patient and automatically alerts the admitting physician of the possibility to screen the patient for eligibility, a method of register-based screening and inclusion that is also used for the first orthopedic rRCT in Sweden, the HipSTHer trial (Wolf et al. 2020). Inclusion criteria are eligibility for a THA according to local guidelines and routines, availability of both treatment options, and signed, informed patient consent (Table 1). Unavailability of both treatment options can be due to implants being out of stock or the lack of the individual surgeon’s competence to use either implant type. Exclusion criteria are cognitive impairment, previous inclusion of a contralateral THA in the ongoing trial, delayed fracture surgery (date of injury more than seven days prior to date of screening), pathological or stress fracture of the femoral neck, and fracture adjacent to a previous ipsilateral hip implant, such as a previously inserted screw or plate.

### Randomization and blinding

Randomization is also performed by use of the study platform incorporated in the SFR. Subjects will be randomized to receive either a DMC (intervention group) or a standard cup (control group), using an allocation sequence hidden from all involved healthcare providers and provided by a trial-independent statistician. There will be no patient or physician blinding.

### Surgical intervention

The trial design is pragmatic, which implies that the choice of implant brands, fixation methods, surgical approach, pre-, peri-, and postoperative routines are based on the participating hospitals’ preferences. Nonetheless, the study protocol requires all participating units to maintain their chosen regime across both intervention and control groups, thus ensuring that only the type of intervention varies per unit. In Sweden, two-thirds of FNF patients who receive a THA are operated on via a direct lateral approach (Swedish Hip Arthroplasty Register 2018) according to Hardinge (1982) or Gammer (1985), and the remaining third via a posterior approach. Surgical approach can vary by surgeon, but individual surgeons must maintain the same approach for both study groups. If the posterior approach is used, the posterior capsule and short external rotators should be repaired, but if individual surgeons choose to abstain from this recommendation, they are free to do so, provided they maintain this regime across treatment groups.

### Implants

#### DMC

The 3 cup brands Avantage (Zimmer Biomet, Warsaw, IN, USA), Polar (Smith & Nephew, London, UK), and Ades (Zimmer Biomet) account for 97% of the DMC used in Swedish FNF patients, and none other than these are currently used at the participating units (Swedish Hip Arthroplasty Register 2018). Should novel DMCs be introduced during the trial period they may be used in study participants, provided that an adequate introduction to the specifics of each implant has been given by the manufacturer. For smaller cup sizes, below 50 mm for the most common DMC, only liners with an inner diameter of 22 mm are available, necessitating the use of 22 mm femoral heads in patients operated on with small cup diameters, whereas 28 mm heads are used in combination with all medium- to large-sized cups.

#### Standard cups

The variation in the use of standard cups in Swedish FNF patients is slightly larger, with the Lubinus (Waldemar Link, Hamburg, Germany), Marathon (DePuy Synthes, Warsaw, IN, USA), Exeter RimFit (Stryker, Kalamazoom MI, USA), and Lubinus IP (Waldemar Link) cups being the most common. As for DMC, the smallest cup sizes require the use of femoral head sizes of 22 mm.

#### Stem components

The Lubinus SP2 (Waldemar Link), Exeter (Stryker), and MS-30 (Zimmer) stems are used in more than 90% of Swedish FNF patients, and the stem type that represents the local standard for FNF patients who receive a THA will be used at each participating unit.

#### Postoperative treatment

Weight bearing will be allowed according to local routines at participating units in both study groups. Postoperative mobilization will start day 0 or 1, which today is the standard of care. Hip precautions can vary between units but must be consistent across groups, ensured by instructions in the study protocol stating that the same educational material, oral information, and rehabilitation regime are presented to all study participants within a given unit, regardless of whether DMC or standard cups were inserted.

#### Withdrawal of patients from the trial

Participants are free to withdraw from the trial at any time without any adverse consequences to further treatment. Already collected data on patients who choose to withdraw their consent to participate in the trial will be retained in the study database, but no additional data including data derived from cross-matching of the SFR database with other registries will be added. Patients who withdraw from the study will not be replaced.

### Endpoints

#### Primary endpoint

The primary endpoint is the occurrence of any dislocation treated with closed or open reduction of the index joint within one year. Dislocation is treated as a binary categoric variable that is registered together with an underlying time-to-event variable. The occurrence of dislocations is determined by linking the study cohort derived from the SFR with the Swedish Hip Arthroplasty Register (SHAR) and the Swedish National Patient Register (NPR). In the SHAR, reoperations and revisions of the index joint are registered, including all open reductions, but excluding closed reductions that are not reported to this register. In the NPR, both closed and open reductions including laterality are registered using International Classification of Diseases (ICD)-10 and NOMESCO codes (Table 2), thus indicating a diagnosis of dislocation and/or its treatment. The presence of a contralateral THA is expected in about 20% of the study participants (Swedish Hip Arthroplasty Register 2018), and to avoid false-positive events due to errors in laterality coding medical charts of all study participants who have been identified as having experienced dislocations will be assessed, and it will thus be ascertained on which joint the reduction or revision procedure was performed.

#### Secondary endpoints

Secondary endpoints are the relative risk of any reoperation of the index joint, PJI of the index joint, and mortality within 90 days and 1 year in the intervention compared with the control group. Any reoperation of the index THA is defined as the occurrence of any surgical procedure performed on the previously treated hip within 1 year after surgery. Reoperations are registered in the SHAR, but the occurrence of reoperations will be additionally verified by cross-matching study participants with the NPR and searching for ICD-10- and NOMESCO-codes (Table 2) indicative of reoperations. A PJI is defined based on the registration of ICD-10 and NOMESCO codes obtained from the NPR (Table 2). Deaths and dates of death are registered in the NPR, allowing for the calculation of 90-day and 1-year mortality. Patient-reported outcomes will be assessed by use of EQ-5D domain score (5 levels) and by the EQ-5D-visual analogue scale (VAS) on a 0–100 numeric scale. Both parameters are routinely collected in the SHAR and will be assessed 1 year after index surgery. Procedural costs for both intervention and control treatment will be recorded at representative sites, as will procedural costs for closed reductions and reoperations.

#### Data collection

Baseline data on age, sex, injury mechanism, fracture classification, time of diagnosis obtained by radiography, and time and type of surgical treatment are transcribed from the SFR to the study database. Answers given by the admitting physician in response to the screening questions will be saved to the study database in order to enable an analysis of reasons underlying the failure to include eligible patients in the trial. Postoperatively, procedural details on the type of surgical approach, type of cup and stem fixation, cement brands, cup and stem brands, cup and femoral head diameter, femoral neck length, and stem size are registered in the SHAR according to national routines. In addition to these procedural details body mass index (BMI) and American Society of Anesthesiologists (ASA) class are recorded in the SHAR.

After trial completion, the study cohort obtained from the SFR will be linked to information on the study participants registered in the SHAR and the NPR, and these data will be entered into a common research database.

#### Data quality assurance

A study monitor will have regular contacts with all participating units in order to (1) verify the presence of informed consent forms signed by participating subjects, (2) confirm that the team at each participating unit adheres to the study protocol, (3) specifically verify that inclusion and exclusion criteria are consistent, and (4) assist locally responsible investigators regarding technical issues with the study platform. A locally responsible study coordinator ensures that all personnel involved in the treatment of trial subjects at each participating unit are adequately informed and trained regarding protocol requirements, and that the standardization of surgery and postoperative treatment across treatment groups is adhered to. The steering committee will have no access to outcomes until the database is locked.

### Estimated sample size and power

#### Scenario 1

For our power calculation, we assume that the 1-year incidence of dislocation after insertion of a standard THA after FNF is 7%, thus slightly lower than the 8% dislocation rate described in Swedish FNF patients treated with a THA (Jobory 2020). For the intervention group operated on with a DMC we assume a relative risk of 0.5, giving an incidence of dislocation of 3.6%. This risk reduction is based on the relative risk of dislocations after the use of a DMC estimated in previous observational studies, ranging from 0.3 to 0.5 (Hailer et al. 2012, Tarasevicius et al. 2013, Bensen et al. 2014, Jobory et al. 2019).

#### Scenario 2

A recent study from Denmark that investigates the use of DMC in FNF patients reports a dislocation rate of 4.7% after a mean follow up of 5.4 years (Tabori-Jensen et al. 2019). To account for this alternative, more pessimistic scenario, we calculate power based on the assumption of a 1-year dislocation rate of 8% in the control group and 4.5% in the intervention group, giving a relative risk of 0.55.

Sample size was determined by simulations under a simplified assumption of a constant risk during the 1-year follow-up, with 25% of the control-arm patients having an event risk of 6.4% (no risk factors), 50% of patients having a 7.4% event risk (one risk factor), and 25% of patients having a 8.5% event risk (two risk factors), corresponding to sex and surgical approach as independent risk factors associated with an increased risk of dislocation (Hailer et al. 2012). Random censoring due to death was assumed to occur exponentially at 10%/year. This assumption is based on a Swedish study on hip fracture patients treated with a THA (Hailer et al. 2016) and is also in line with mortality data in patients treated with THA due to FNF that is reported by the SHAR (Swedish Hip Arthroplasty Register 2018).

With a sample size of n = 1,600 patients, the trial has 88% power to detect a reduction in 1-year dislocation rates from 7% to 3.6%, equaling a hazard ratio of 0.5 (scenario 1), and 83% power to detect a reduction from 8% to 4.5%, equaling a hazard ratio of 0.55 (scenario 2).

### Statistics

Analysis will be performed using the intention-to-treat principle including all randomized patients according to randomized treatment. The primary outcome is the adjusted risk of dislocation treated by open or closed reduction within 1 year. The cumulative unadjusted incidence of dislocations will be estimated using the Kaplan–Meier method per randomized treatment group. The relative hazard of dislocation in the intervention compared with the control group will be estimated by Cox regression models adjusted for sex and surgical approach and will be presented as a hazard ratio with 95% profile likelihood confidence interval and a two-sided likelihood-ratio p-value. With the registry-nested follow-up, we assume that follow-up will be complete, but in the rare case that a patient has incomplete follow-up he or she will be considered censored at last known follow-up. Death before dislocation will be handled as censoring at day of death.

The secondary endpoints any reoperation, PJI, and mortality will be analyzed and described in the same way as the primary endpoint. Supplementary sensitivity analyses will be performed for all event endpoints. These analyses will primarily use logistic regression with the same covariates as the primary analysis, and as a supplement risk differences with Wald confidence intervals will be computed. To investigate sensitivity to baseline covariates, unadjusted Cox regression models will be fitted. Sensitivity analyses to investigate the impact of censoring by death, in addition to analyzing death as an outcome, will include analyses of the composite of dislocation and death performed similarly to the primary endpoint analysis. Estimation of the risk of dislocation after 1 year will be investigated in an additional sensitivity analysis including patients with follow-up exceeding 1 year. Randomized and actual treatments will be described in a CONSORT diagram, and additional per-protocol analyses will be undertaken as sensitivity analyses. The threshold of statistical significance will be set at a two-sided p-value of 0.05. Secondary endpoints will be presented without formal multiplicity adjustment.

EQ-5D domain scores (5 levels) at 1 year after index surgery will be summarized using descriptive frequency tables by randomized treatment. They will be analyzed by using proportional odds logistic regression adjusted for the baseline domain score as a categorical variable and presented as the common odds ratio for all cut-points. For the primary presentation and analysis, missing domain scores due to death will be considered a separate category. For the adjusted analysis, missing baseline scores will be imputed using multiple imputation. Sensitivity analyses using observed cases only will also be provided.

EQ-5D VAS score at 1 year after index surgery will be presented using tables of medians and quartiles as well as empirical cumulative distribution plots of VAS score and linear change in VAS from baseline. The VAS score will be analyzed using proportional odds logistic regression adjusted baseline score as a numerical variable modelled as a restricted cubic spline. Missing baseline scores will be imputed using multiple imputation. Outcome scores that are missing due to death will primarily be imputed as 0, with no imputation of other missing scores.

For all event outcome variables, pre-defined subgroup/interaction analyses to assess the homogeneity of the treatment contrast will be performed for sex, age, ASA class, and BMI, and for the procedural characteristics femoral neck length, cup diameter, femoral head diameter, type of cup, type of stem, type of cement, and surgical approach. For categorical subgroup indicators, events will be described in each subgroup as for the entire population, and the treatment contrast in each subgroup will be estimated using a Cox proportional hazard model with treatment, subgroup, indicator, and interaction, and presented with nominal 95% confidence intervals for each subgroup and the interaction p-value. For age, sex, and BMI, the interaction model will use restricted cubic spline modelling, and present the results as a curve of treatment contrast by covariate with 95% pointwise confidence bands and the interaction p-value. Treatment comparison is not relevant for subgroups that are specific to a single treatment arm. For such subgroups descriptive statistics including Kaplan–Meier plots will be presented for each subgroup. For health economic studies, Markov modelling based on the assumption of defined health states will be performed, and the primary outcome will be cost per quality-adjusted life year. Deterministic and probabilistic sensitivity analyses of the main model hypothesis and variables will be performed in addition to the main analyses.

## Ethics, registration, data sharing plan, funding, potential conflicts of interests, and dissemination

The study is performed in accordance with the published study protocol, with the latest version of the Declaration of Helsinki, and applicable regulatory requirements. The study was approved by the Swedish Ethical Review Authority (Approval No: 2019-01137). Patients will be required to give written informed consent to participate.

The trial is registered at clinicaltrials.gov (NCT03909815; December 12, 2019).

Datasets derived from the current study that are needed to replicate main findings will be made available by the principal investigators upon reasonable request.

The trial is supported by a grant from the Swedish Research Council (VR 2019-00436). The funding body has no authority over study design, data collection management, interpretation of data, analysis, or writing of manuscripts. The formal sponsor following the definition of clinicaltrials.gov is Uppsala University, Sweden. Open access funding is provided by Uppsala University.

NPH reports both institutional support and lecturer’s fees from Waldemar Link GmbH and Zimmer Biomet, 2 manufacturers of DMCs used in this study. OW reports lecturer’s fees from Waldemar Link GmbH, Smith & Nephew and DePuy Synthes. MM reports lecturer’s fees from DePuy Synthes. None of the other authors declare any conflict of interest.

The results from the study the will be distributed through presentions and publication in a scientific peer-review medical journal.

### Study start and duration

The first patient was recruited on January 9, 2020. We expect to recruit patients for 5 years. 

## Discussion

The purpose of the DUALITY trial is to provide evidence to support or refute the use of DMC in patients with a displaced FNF treated with THA. The potentially reduced risk of dislocation after the use of DMC in FNF patients is described in several observational studies (Tarasevicius et al. 2013, Bensen et al. 2014, Tabori-Jensen et al. 2019), but no high-level evidence study has yet been conducted (Griffin et al. 2016), and there may be an increased risk of other adverse events such as loosening or PJI (Kreipke et al. 2019). The number of elderly patients with displaced FNF treated with THA is increasing in most developed countries, but prior to the broad introduction of the costlier DMC concept its safety and efficacy, including cost-effectiveness from a health-economic perspective, must be evaluated (Horriat and Haddad 2018, Bernstein et al. 2019).

### Strengths and limitations

The obvious weakness of previous observational studies is the presence of residual confounding, and confounding by indication may be introduced by the fact that DMC may have been preferentially used in patients who were at higher risk of dislocation. Other limitations to previous studies include small sample sizes or the lack of comparison groups (Adam et al. 2012, Tabori-Jensen et al. 2019). Thus, by conducting a large-scale RCT, we investigate a sufficiently powered sample of patients randomized to intervention or control treatment, thereby reducing problems related to residual confounding or insufficient sample size. Additionally, the lack of external validity inherent in classical RCT designs may be improved by the pragmatic study design of our trial: broad inclusion criteria, few exclusion criteria, freedom to choose locally established implant brands, surgical approach, and postoperative restrictions contribute to the generalizability of our future findings.

The SFR, supplying the platform used for screening, inclusion, and randomization of our study cohort, is a population-based register of all fractures in adults and long-bone fractures in children, regardless of treatment (Wennergren et al. 2015). Linkage of the study cohort with the SHAR and the NPR is performed to gain access to additional baseline data, procedural details, and primary and most secondary outcomes. The SHAR has completeness of 96–98% and 100% coverage (Swedish Hip Arthroplasty Register 2018), completeness for the NPR is above 99%, and its positive predictive value is 85–95% (Ludvigsson et al. 2011). Thus, we believe our data sources to be valid and reliable. Nonetheless, optimal control over primary outcome necessitates individual medical chart assessment of all study participants who are registered with a dislocation in order to ascertain correct laterality and diagnosis.

There are numerous potential limitations:

1. The assumptions underlying the sample size calculation are key to every RCT, and we have attempted at calculating 2 realistic scenarios. Importantly, the dislocation rate of 8% after conventional THA in patients with FNF is based on a recent Swedish study (Jobory 2020). The risk reduction of 0.5 that we assume is associated with the use of DMC cups in the main scenario 1 is based on several observational studies, and this number is at the upper end of the range of reported risk reductions, thus pessimistic. The alternative scenario 2 is based on a recent Danish study (Tabori-Jensen et al. 2019) reporting a higher dislocation rate, which may be explained by the following factors: (a) The longer follow-up period in that study may lead to a higher incidence of dislocations when compared with the 1-year incidence of dislocations that our main scenario is based upon. (b) All patients in the Danish study were operated on via a posterior approach that is known to be associated with an increased risk of dislocation (Hailer et al. 2012), whereas two-thirds of Swedish patients receiving a THA due to a femoral neck fracture are operated on via direct lateral approaches (Swedish Hip Arthroplasty Register 2018). (c) More than half of the Danish cohort were treated with cementless implants, a choice that is also associated with an increased risk of dislocation (Chammout et al. 2017). Nonetheless, our sample size lends above 80% power to detect a relevant difference between the intervention and control group, even in this alternative scenario.

2. We believe that we can include a sample of 1,600 patients within reasonable time, mainly based on the participation of at least 14 orthopedic units that together performed about 2,200 THA procedures on patients with FNF during the period 2016–2018, and more units are expected to be enrolled in the near future. However, several factors can delay inclusion: (a) There is a national recommendation to treat patients with FNF within 24 hours of admission to hospital, thus the time window for screening, inclusion, and randomization is limited and may be too short. (2) Surgical expertise to perform either intervention or control treatment is required but not always available, resulting in failure to include patients, or in cross-over if patients allocated to one treatment receive the other. (3) Cognitive impairment is present in a large proportion of FNF patients. At some participating units such patients receive THA, but these will not be included because the ethical approval was restricted to cognitively intact patients. (4) Last but not least, acute and unforeseen events with a large impact on the available resources in healthcare systems, such as the COVID-19 pandemic, can jeopardize any prospective study, and the effects of the current situation on the inclusion of patients in our trial are already dramatic.

3. Confounding factors such as sex, age, ASA class, BMI, femoral neck length, cup diameter, femoral head diameter, type of cup, type of stem, type of cement, and surgical approach are not stratified for in our trial, and, in a worst-case scenario, these confounders may be unevenly distributed across trial arms. We attempt to address this potential issue by adjusting for the main effect mediators sex and surgical approach, and will undertake subgroup and interaction analyses for all variables mentioned.

In summary, the proposed RCT with its register-nested, pragmatic design will, it is hoped, provide high-level evidence on the topic of DMC in patients with displaced FNF.

NPH, OW, MM, and SM designed the trial and shared in reviewing the manuscript. Ethical applications were handled by NPH. MN drafted the manuscript. All authors have given their final approval of the version to be published and agree to be accountable for all aspects of the work.

The authors thank Krister Arlinger for his valuable contributions to the study protocol, and Krister Arlinger and Fredrik Heidgert for the technical solutions behind the study platform in the SFR. They gratefully acknowledge the financial support of the Swedish Research Council and logistic support from Uppsala Clinical Research Center, especially statistician Ollie Östlund, who performed power calculations and drafted the statistical analysis plan. They thank study monitor Monica Sjöholm for invaluable help with all practical issues of this trial. The support of Gothia Forum, the Center of Registers at the Western Healthcare Region, and the authors’ respective institutions and departments is gratefully acknowledged.

**Table 1.** Screening questions within the SFR platform

This patient is eligible for inclusion in the Duality trial for randomization to receive a standard cup or a dual mobility cup for a Garden 3–4 fracture. Answer the following questions for screening.
Is the patient already treated for the fracture? Is the patient suitable for a total hip arthroplasty? Can both treatments (standard and dual mobility cup) be performed for this patient? • Has the patient given informed consent?

**Table 2.** ICD-10 and NOMESCO codes defining primary and secondary endpoints

Endpoint	Codes
**ICD**	
Dislocation Periprosthetic joint infection	M24.3, M24.4, M24.4F, S73.0, T93.3 M00.0, M00.0F, M00.1, M00.2, M00.2F, M00.8, M00.8F, M00.9, M00.9F, M86.0F, M86.1F, M86.6, M86.6F, T81.4, T84.5, T84.5F, T84.5X, T84.7, T84.7F
	
**NOMESCO**	
Dislocation Periprosthetic joint infection Any reoperation	NFH00, NFH02, NFH20, NFH21, NFH22 NFSx, NFA12, TNF05, TNF10
	Any of the codes above, and: NFA00-22, NFA31-32, NFCx, NFF01–12, NFL09–19, NFL39–49, NFL69–99, NFM09–29, NFM49, NFM79–99, NFTx, NFWx

## References

[CIT0001] Adam P, Philippe R, Ehlinger M, Roche O, Bonnomet F, Mole D, Fessy M H, French Society of Orthopaedic S Traumatology. Dual mobility cups hip arthroplasty as a treatment for displaced fracture of the femoral neck in the elderly: a prospective, systematic, multicenter study with specific focus on postoperative dislocation. Orthop Traumatol Surg Res 2012; 98(3): 296–300. 22463868 10.1016/j.otsr.2012.01.005

[CIT0002] Bensen A S, Jakobsen T, Krarup N. Dual mobility cup reduces dislocation and re-operation when used to treat displaced femoral neck fractures. Int Orthop 2014; 38(6): 1241–5. 24441666 10.1007/s00264-013-2276-8 PMC4037505

[CIT0003] Bernstein J, Weintraub S, Morris T, Ahn J. Randomized controlled trials for geriatric hip fracture are rare and underpowered: a systematic review and a call for greater collaboration. J Bone Joint Surg Am 2019; 101(24): e132. 31567688 10.2106/JBJS.19.00407

[CIT0004] Bhandari M, Swiontkowski M. Management of acute hip fracture. N Engl J Med 2017; 377(21): 2053–62. 29166235 10.1056/NEJMcp1611090

[CIT0005] Caton J H, Ferreira A. Dual-mobility cup: a new French revolution. Int Orthop 2017; 41(3): 433–7. 28197702 10.1007/s00264-017-3420-7

[CIT0006] Caton J H, Prudhon J L, Ferreira A, Aslanian T, Verdier R. A comparative and retrospective study of three hundred and twenty primary Charnley type hip replacements with a minimum follow up of ten years to assess whether a dual mobility cup has a decreased dislocation risk. Int Orthop 2014; 38(6): 1125–9. 24737147 10.1007/s00264-014-2313-2 PMC4037498

[CIT0007] Chammout G, Muren O, Laurencikas E, Boden H, Kelly-Pettersson P, Sjoo H, Stark A, Skoldenberg O. More complications with uncemented than cemented femoral stems in total hip replacement for displaced femoral neck fractures in the elderly. Acta Orthop 2017; 88(2): 145–51. 27967333 10.1080/17453674.2016.1262687 PMC5385108

[CIT0008] Conroy J L, Whitehouse S L, Graves S E, Pratt N L, Ryan P, Crawford R W. Risk factors for revision for early dislocation in total hip arthroplasty. J Arthroplasty 2008; 23(6): 867–72. 18534522 10.1016/j.arth.2007.07.009

[CIT0009] Cuthbert R, Wong J, Mitchell P, Kumar Jaiswal P. Dual mobility in primary total hip arthroplasty: current concepts. EFORT Open Rev 2019; 4(11): 640–6. 31754471 10.1302/2058-5241.4.180089 PMC6851525

[CIT0010] Darrith B, Courtney P M, Della Valle C J. Outcomes of dual mobility components in total hip arthroplasty: a systematic review of the literature. Bone Joint J 2018; 100-B(1): 11–9. 29305445 10.1302/0301-620X.100B1.BJJ-2017-0462.R1

[CIT0011] Devane P A, Wraighte P J, Ong D C, Horne J G. Do joint registries report true rates of hip dislocation? Clin Orthop Relat Res 2012; 470(11): 3003–6. 22451337 10.1007/s11999-012-2323-6 PMC3462862

[CIT0012] Enocson A, Pettersson H, Ponzer S, Tornkvist H, Dalen N, Tidermark J. Quality of life after dislocation of hip arthroplasty: a prospective cohort study on 319 patients with femoral neck fractures with a one-year follow-up. Qual Life Res 2009a; 18(9): 1177–84. 19714486 10.1007/s11136-009-9531-x

[CIT0013] Enocson A, Hedbeck C J, Tidermark J, Pettersson H, Ponzer S, Lapidus L J. Dislocation of total hip replacement in patients with fractures of the femoral neck. Acta Orthop 2009b; 80(2): 184–9. 19404800 10.3109/17453670902930024 PMC2823165

[CIT0014] Gammer W. A modified lateroanterior approach in operations for hip arthroplasty. Clin Orthop Relat Res 1985; (199): 169–72. 4042474

[CIT0015] Griffin X L, Parsons N, Achten J, Costa M L. A randomised feasibility study comparing total hip arthroplasty with and without dual mobility acetabular component in the treatment of displaced intracapsular fractures of the proximal femur: the Warwick Hip Trauma Evaluation Two: WHiTE Two. Bone Joint J 2016; 98-B(11): 1431–5. 27803216 10.1302/0301-620X.98B11.BJJ-2016-0478.R1

[CIT0016] Hailer N P, Weiss R J, Stark A, Kärrholm J. The risk of revision due to dislocation after total hip arthroplasty depends on surgical approach, femoral head size, sex, and primary diagnosis: an analysis of 78,098 operations in the Swedish Hip Arthroplasty Register. Acta Orthop 2012; 83(5): 442–8. 23039167 10.3109/17453674.2012.733919 PMC3488169

[CIT0017] Hailer N P, Garland A, Rogmark C, Garellick G, Kärrholm J. Early mortality and morbidity after total hip arthroplasty in patients with femoral neck fracture. Acta Orthop 2016; 87(6): 560–6. 27649030 10.1080/17453674.2016.1234869 PMC5119437

[CIT0018] Hardinge K. The direct lateral approach to the hip. J Bone Joint Surg Br 1982; 64(1): 17–19. 7068713 10.1302/0301-620X.64B1.7068713

[CIT0019] Horriat S, Haddad F S. Dual mobility in hip arthroplasty: what evidence do we need? Bone Joint Res 2018; 7(8): 508–10. 30258569 10.1302/2046-3758.78.BJR-2018-0217 PMC6138808

[CIT0020] James S, Rao S V, Granger C B. Registry-based randomized clinical trials: a new clinical trial paradigm. Nat Rev Cardiol 2015; 12(5): 312–6. 25781411 10.1038/nrcardio.2015.33

[CIT0021] Jobory A. Dislocation after hip fracture related arthroplasty: incidence, risk factors and prevention. Thesis, Lund University; 2020.

[CIT0022] Jobory A, Kärrholm J, Overgaard S, Becic Pedersen A, Hallan G, Gjertsen J E, Makela K, Rogmark C. Reduced revision risk for dual-mobility cup in total hip replacement due to hip fracture: a matched-pair analysis of 9,040 cases from the Nordic Arthroplasty Register Association (NARA). J Bone Joint Surg Am 2019; 101(14): 1278–85. 31318807 10.2106/JBJS.18.00614

[CIT0023] Johansson T, Jacobsson S A, Ivarsson I, Knutsson A, Wahlstrom O. Internal fixation versus total hip arthroplasty in the treatment of displaced femoral neck fractures: a prospective randomized study of 100 hips. Acta Orthop Scand 2000; 71(6): 597–602. 11145387 10.1080/000164700317362235

[CIT0024] Jolles B M, Zangger P, Leyvraz P F. Factors predisposing to dislocation after primary total hip arthroplasty: a multivariate analysis. J Arthroplasty 2002; 17(3): 282–8. 11938502 10.1054/arth.2002.30286

[CIT0025] Kazley J M, Banerjee S, Abousayed M M, Rosenbaum A J. Classifications in brief: Garden classification of femoral neck fractures. Clin Orthop Relat Res 2018; 476(2): 441–5. 29389800 10.1007/s11999.0000000000000066 PMC6259691

[CIT0026] Kim Y H, Choi Y, Kim J S. Influence of patient-, design-, and surgery-related factors on rate of dislocation after primary cementless total hip arthroplasty. J Arthroplasty 2009; 24(8): 1258–63. 19896063 10.1016/j.arth.2009.03.017

[CIT0027] Ko L M, Hozack W J. The dual mobility cup: what problems does it solve? Bone Joint J 2016; 98-B(1 Suppl. A): 60–3. 26733643 10.1302/0301-620X.98B1.36332

[CIT0028] Kreipke R, Rogmark C, Pedersen A B, Kärrholm J, Hallan G, Havelin L I, Makela K, Overgaard S. Dual mobility cups: effect on risk of revision of primary total hip arthroplasty due to osteoarthritis: a matched population-based study using the Nordic Arthroplasty Register Association database. J Bone Joint Surg Am 2019; 101(2): 169–76. 30653047 10.2106/JBJS.17.00841

[CIT0029] Ludvigsson J F, Andersson E, Ekbom A, Feychting M, Kim J L, Reuterwall C, Heurgren M, Olausson P O. External review and validation of the Swedish national inpatient register. BMC Public Health 2011; 11: 450. 21658213 10.1186/1471-2458-11-450 PMC3142234

[CIT0030] Meek R M, Allan D B, McPhillips G, Kerr L, Howie C R. Epidemiology of dislocation after total hip arthroplasty. Clin Orthop Relat Res 2006; 447: 9–18. 16672897 10.1097/01.blo.0000218754.12311.4a

[CIT0031] Philippot R, Boyer B, Farizon F. Intraprosthetic dislocation: a specific complication of the dual-mobility system. Clin Orthop Relat Res 2013; 471(3): 965–70. 23054529 10.1007/s11999-012-2639-2 PMC3563829

[CIT0032] Phillips C B, Barrett J A, Losina E, Mahomed N N, Lingard E A, Guadagnoli E, Baron J A, Harris W H, Poss R, Katz J N. Incidence rates of dislocation, pulmonary embolism, and deep infection during the first six months after elective total hip replacement. J Bone Joint Surg Am 2003; 85(1): 20–6. 10.2106/00004623-200301000-00004 12533567

[CIT0033] Skoldenberg O, Ekman A, Salemyr M, Boden H. Reduced dislocation rate after hip arthroplasty for femoral neck fractures when changing from posterolateral to anterolateral approach. Acta Orthop 2010; 81(5): 583–7. 20860452 10.3109/17453674.2010.519170 PMC3214747

[CIT0034] Swedish Hip Arthroplasty Register. Annual Report 2017; 2018. Available from: http://registercentrum.blob.core.windows.net/shpr/r/Eng_Arsrapport_2017_Hoftprotes_final-Syx2fJPhMN.pdf.

[CIT0035] Tabori-Jensen S, Frolich C, Hansen T B, Bovling S, Homilius M, Stilling M. Higher UHMWPE wear-rate in cementless compared with cemented cups with the Saturne(R) Dual-Mobility acetabular system. Hip Int 2018; 28(2): 125–32. 29890909 10.1177/1120700018768615

[CIT0036] Tabori-Jensen S, Hansen T B, Stilling M. Low dislocation rate of Saturne((R))/Avantage((R)) dual-mobility THA after displaced femoral neck fracture: a cohort study of 966 hips with a minimum 1.6-year follow-up. Arch Orthop Trauma Surg 2019; 139(5): 605–12. 30547264 10.1007/s00402-018-3093-8

[CIT0037] Tarasevicius S, Robertsson O, Dobozinskas P, Wingstrand H. A comparison of outcomes and dislocation rates using dual articulation cups and THA for intracapsular femoral neck fractures. Hip Int 2013; 23(1): 22–6. 23397197 10.5301/HIP.2013.10632

[CIT0038] Wennergren D, Ekholm C, Sandelin A, Moller M. The Swedish Fracture Register: 103,000 fractures registered. BMC Musculoskelet Disord 2015; 16: 338. 26546157 10.1186/s12891-015-0795-8 PMC4636773

[CIT0039] Wolf O, Sjoholm P, Hailer N P, Moller M, Mukka S. Study protocol: HipSTHeR—a register-based randomised controlled trial—hip screws or (total) hip replacement for undisplaced femoral neck fractures in older patients. BMC Geriatr 2020; 20(1): 19. 31964340 10.1186/s12877-020-1418-2 PMC6975074

